# Genome Sequence of Cluster W Mycobacteriophage Taptic

**DOI:** 10.1128/genomeA.01606-16

**Published:** 2017-03-16

**Authors:** Catherine M. Mageeney, Emily R. Seier, Elise C. Esposito, Lee H. Graham, Emily L. Heckman, Chelsea M. Hipwell, Allison B. Kelliher, Nicole A. Lando, Patricia Y. Morales, Daniel A. Russell, Barbara E. Tsaousis, Margaret A. Kenna, Vassie C. Ware

**Affiliations:** a Department of Biological Sciences, Lehigh University, Bethlehem, Pennsylvania, USA; b Department of Biological Sciences, University of Pittsburgh, Pittsburgh, Pennsylvania, USA

## Abstract

The Taptic genome is the first to be annotated from the W cluster of mycobacteriophages infecting *Mycobacterium smegmatis* mc^2^155. All 92 predicted open reading frames (ORFs) and a single tRNA specifying glycine (tRNA-gly) are transcribed rightward. Many functionally uncharacterized ORFs appear to be W cluster specific, as nucleotide similarity is shared only with other W cluster genomes.

## GENOME ANNOUNCEMENT

Structural and functional annotation of a large collection of mycobacteriophage genomes has revealed extensive genetic diversity in this group, often uncovering novel genes and differences in genome organization that are unique to specific phage types (1). The discovery of new gene products and their role in the phage life cycle within mycobacterial hosts may lead to the development of novel molecular tools to control mycobacterial pathogenesis.

Mycobacteriophage Taptic was isolated on *Mycobacterium smegmatis* mc^2^155 by direct isolation from soil located in Northampton, PA. Taptic produces 0.1- to 0.2-mm clear plaques that appear after 48 h on* M. smegmatis* mc^2^155. Purification, amplification, and electron microscopy of phage particles revealed a *Siphoviridae* morphology, with a head (70 nm)-to-tail (275 nm) ratio of approximately 1 to 4. Genomic DNA was isolated and sequenced by Illumina to 70× coverage and assembled with Newbler and Consed. The genome is 60,973 bp and circularly permuted, as indicated by a lack of read start buildups or substantial regions of above-average coverage. Taptic is a member of cluster W. Currently, two other members have been sequenced and verified as W cluster members (http://www.phagesdb.org).

DNA Master (http://cobamide2.bio.pitt.edu/) was used for genome annotation. Glimmer and GeneMark, embedded in DNA Master, were used to call 92 ORFs. BLASTp and HHpred (2) were used for functional annotation, and Phamerator (3) was used for comparative genomic analysis. Aragorn and tRNAscan (4, 5) identified one tRNA specifying glycine, with anticodon TCC. Putative functions are called for only 21.7% (20 of 92) of the gene products. Consistent with the gene order in other mycobacteriophage genomes (1), the Taptic head and tail genes are positioned in the left half, and DNA replication/maintenance functions encoding endonucleases and recombinases are among the genes in the right half. Genes specifying helix-turn-helix (HTH) DNA binding domain proteins are also among ORFs in the left half of the genome. Very few functions are predicted at the right end, but extensive nucleotide similarity with other verified W cluster phages is apparent. About 40% (37 of 92) of Taptic ORFs belong to W-specific phamilies (phams) of related sequences, as defined by Phamerator (3). Two ORFs (*gp77* and *gp89*) define phams with no known counterparts in other mycobacteriophage genomes (designated orphams). All ORFs are rightward transcribed, an unusual feature not found in most mycobacteriophage genomes, except in clusters H and U (http://www.phagesdb.org; Phamerator [3]).

Taptic produces clear plaques, typically indicative of an obligately lytic phage; yet, bacterial overgrowth within the zone of clearing after extended incubation may suggest lysogenic capability. *gp38* encodes a protein containing a classic repressor HTH DNA binding domain but lacks sequences expected for a completely functional repressor. No integrase is predicted using BLASTP or HHpred (2). Several chromosomal partitioning proteins are predicted with HHpred but have low probabilities (<90%) and/or positive E values (>0.0). *gp74* encodes a ParB homologue; however, without an encoded ParA partner, a canonical chromosomal partitioning mechanism cannot be predicted (6). Whether or not Taptic is a temperate phage remains to be determined.

### Accession number(s).

The Taptic genome sequence is available in GenBank with accession number KY130461.
